# Identification of a class of potent USP25/28 inhibitors with broad-spectrum anti-cancer activity

**DOI:** 10.1038/s41392-022-01209-2

**Published:** 2022-12-08

**Authors:** Jin Peng, Kun Jiang, Xiao Sun, Lingzhi Wu, Jiewei Wang, Xiaomei Xi, Xu Tan, Tingbo Liang, Changheng Tan, Pumin Zhang

**Affiliations:** 1 Zhejiang Provincial Key Laboratory of Pancreatic Diseases, The First Affiliated Hospital of Zhejiang University, Hangzhou, Zhejiang 310003 China; 2 Shanghai Institute of Materia Medica, Shanghai, 200216 China; 3 Chaser Therapeutics, Inc., Hangzhou, Zhejiang Province 310018 China; 4 Beijing Advanced Innovation Center for Structural Biology, School of Pharmaceutical Sciences, Tsinghua-Peking Center for Life Sciences, Tsinghua University, Beijing, China

**Keywords:** Target validation, Molecular medicine

**Dear Editor**,

An increasing body of evidence suggests that USP28 could be a target for cancer treatment^1–4^. To identify its inhibitors, we screened a 100-thousand synthetic compound library and found 3 lead compounds, CT1001-1003, that showed significant inhibitory activity (Supplementary Fig. 1a). The optimization of these compounds led to CT1018, a much stronger inhibitor (Supplementary Fig. 1b). CT1018 is almost the same as CT1002 except that it is semi-methylated at the 3-amino group. Interestingly, fully methylating the 3-amino group (CT1038, Supplementary Fig. 1c) almost killed the inhibitory activity, and replacing the methyl group with an ethyl group (CT1047, Supplementary Fig. 1c) also led to a weaker inhibitor. These results indicate that the semi-methylation of the 3-amino group is critical for the inhibitory activity. However, CT1018 was inactive in cell-based assays. Continued optimization resulted in CT1073 and CT1113 (Fig. 1a). These two compounds are potent against USP28 as well as the closely-related USP25 (Supplementary Fig. 1f). The importance of the semi-methylation at the 3-amino group is also true for CT1073, as its unmethylated version, CT1008, is a much weaker inhibitor (Supplementary Fig. 1d). CT1113 contains a chiral center and one enantiomer is much more potent than another (Supplementary Fig. 1e). The specificity of these compounds was demonstrated by the lack of activities of as much as 10 μM CT1073 or CT1113 against other deubiquitinases and SENP1 (Supplementary Fig. 1g). Furthermore, we measured the interaction kinetics between the inhibitors and USP25/28 with an SPR instrument. As shown in Supplementary Fig. 1h, i, CT1073 and CT1113 have similar KDs for USP25 and USP28.

**Fig. 1:**
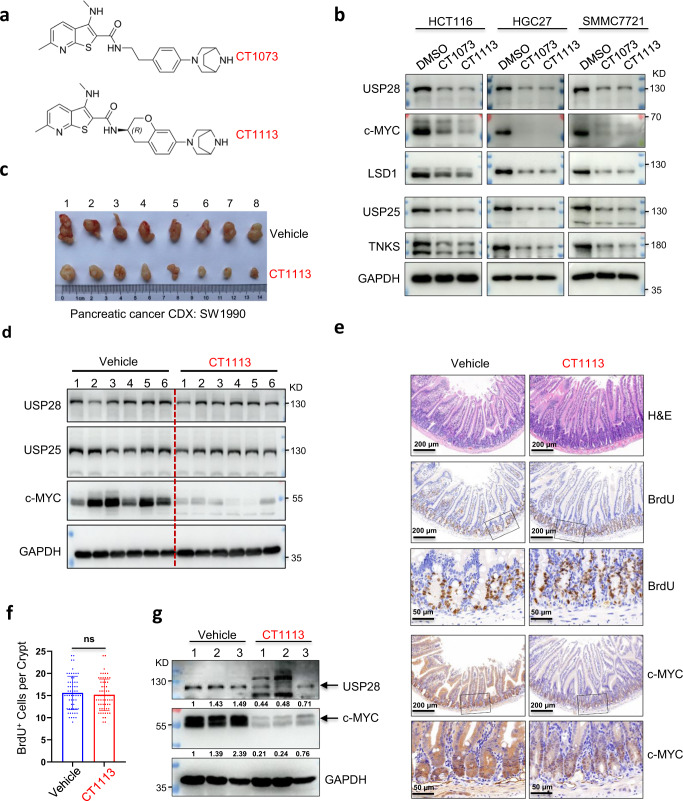
Identification of USP28 inhibitors. **a** The chemical structures of CT1073 and CT1113. **b** Western blotting analysis of the indicated proteins in the cells treated with 500 nM CT1073 or CT1113 for 24 h. **c** The effect of CT1113 in a pancreatic cancer CDX model generated by inoculating SW1990 cells into Balb/c nude mice (*n* = 8 mice per group). The photographs of the tumors are presented. **d** Western blotting analysis of the tumor samples shown in **c**. **e** Hematoxylin and eosin (H&E) staining and immunohistochemical staining of the small intestines from the vehicle control and CT1113 treated animals (*n* = 3 mice per group). **f**. Quantitation of BrdU incorporation. The BrdU positive cells in the intestinal crypts (**e**) were counted and plotted. Student’s *t-*test: n.s. indicates no significant difference. **g** Western blotting analysis of USP28 and c-MYC levels in the intestine from control and CT1113-treated mice. The numbers are band intensities of USP28 or c-MYC relative to GAPDH which were further normalized against sample 1 in the control group

Next, we subjected USP25/28 inhibitors to various cell-based assays. First, we examined their effectiveness in inhibiting the DUBs in cells. c-MYC is the most notable substrate of USP28^5^. As shown in Supplementary Fig. 2a, both CT1073 and CT1113 were able to reduce c-MYC levels quite dramatically in a diverse set of cancer cell lines and the reduction occurred within 1–2 h of the treatment. As expected, the expression of MYC-MAX target genes was greatly suppressed in the treated cells (Supplementary Fig. 2b). With a longer treatment time, we could see dramatic decreases in the levels of USP28 and USP25 themselves and their substrates LSD1 and Tankyrase (TNKS) (Fig. 1b). In a cycloheximide chase experiment, we could see clearly the destabilization of c-MYC by CT1073 treatment (Supplementary Fig. 2c, d), and a further ubiquitination assay demonstrated that CT1073 or CT1113 treatment resulted in more ubiquitination of c-MYC protein (Supplementary Fig. 2e, f) and more ubiquitination of Tankyrase (Supplementary Fig. 2g). As expected, the half-lives of LSD1 and TNKS were all shortened by CT1073 treatment (Supplementary Fig. 2h, i) and the destabilization of c-MYC, LSD1, and TNKS caused by CT1073 or CT113 treatment could be reversed by the proteasome inhibitor MG132 (Supplementary Fig. 2j). In addition, we examined the effect of CT1113 on the protein levels of p53 and CHK2. No significant destabilization of these two proteins was observed (Supplementary Fig. 2k). Together, these data demonstrate that the USP25/28 inhibitors we developed are effective in cells.

We next determined the EC_50_s of CT1073 and CT1113 against the proliferation or cell viability of a diverse panel of tumor cell lines. As shown in Supplementary Fig. 3a, both compounds were very effective in suppressing the proliferation of these cells. The terminal phenotype of CT1073-treated cancer cells is either apoptosis (Supplementary Fig. 3b) or cell cycle arrest (Supplementary Fig. 3c). Interestingly, the cell cycle arrests occurred in G2 and even S phase (Supplementary Fig. 3c). These results suggest that most, if not all, cancer cells require *USP25* and/or *USP28* for proliferation and/or survival. To test that genetically, we depleted the expression of *USP25*, *USP28*, or both in selected cancer cell lines. In T47D, the depletion of either *USP25* or *USP28* imposed some suppression of proliferation, but the depletion of both resulted in a blockade of proliferation and eventual cell death (Supplementary Fig. 3d). In A549, depleting *USP25* or *USP28* alone was sufficient to suppress the proliferation, and the double depletion resulted in cell death (Supplementary Fig. 3d). Moreover, we overexpressed *USP25* and *USP28*, either singularly or together in HCT116 cells. The overexpression of *USP25* or *USP28* alone did not alter the sensitivity of the cells to CT1113 much, but the overexpression of both simultaneously reduced the sensitivity (Supplementary Fig. 3e), increasing the EC_50_ from 65 nM in GFP-expressing control cells to 92 nM in the *USP25/28* overexpression cells. These data suggest that the anti-proliferative effect of CT1113 is largely an on-target effect, although the contributions from potential off-target effects could not be completely excluded.

We next wanted to demonstrate their effectiveness in vivo. CT1073 could not be given orally and was metabolically unstable in mice, most likely because it contains an easily hydrolysable amide bond. CT1113 (Fig. 1a) had the amide bond modified to overcome that and were used for in vivo anti-tumor studies. We first tested it against xenograft tumors formed by SW1990, a human pancreatic cancer cell line. As shown in Fig. 1c and Supplementary Fig. 4a, CT1113 treatment resulted in significant suppression of the tumor growth. The compound also caused MYC levels to decrease (Fig. 1d). Ki67 staining demonstrated much reduced proliferation in CT1113 treated tumors than that in the vehicle controls (Supplementary Fig. 4b). The treated animals looked normal, except they seemed not consume as much food as the control animals, which was likely the cause of the decreases in their body weight (Supplementary Fig. 4c). Next, we tested CT1113 on a colon cancer CDX model using the colon cancer cell line HCT116. Similar efficacy was observed (Supplementary Fig. 4d). Taken together, these in vivo results demonstrate that USP25/28 inhibitor CT1113 is a strong anti-tumor agent.

Since c-MYC is important for the proliferation of normal cells as well, we wanted to examine the effects of CT1113 on proliferative tissues in mice, especially the intestine which was shown to be dependent on *MYC* for regeneration^6^. A group of normal C57BL/6 mice were given CT1113 for 21 days and recovered for another 21 days (Supplementary Fig. 5a). At the end of the dosing scheme, the animals did not display any noticeable signs of toxicities, although their body weight decreased about 10% by the end of dosing (Supplementary Fig. 5b), as we saw in tumor-bearing nude mice (Supplementary Fig. 4c). However, the body weight picked up quickly upon stopping the administration of CT1113 (Supplementary Fig. 5b). Histological examination of major organs did not reveal any significant changes either. In the intestine, the villi were indistinguishable in the CT1113-treated mice from that in control animals (Fig. 1e). Surprisingly, the proliferation in the crypts (measured by BrdU incorporation) was undisrupted at all by CT1113 treatment (Fig. 1e, f). Immunohistochemical staining of c-MYC showed very strong c-MYC expression in the control but greatly reduced in the CT1113-treated intestine (Fig. 1e), consistent with the result from the western blotting analysis (Fig. 1g). However, c-MYC expression persisted in the crypts (Fig. 1e), indicating that the stem cells could maintain c-MYC expression despite the inhibition of USP28. This result provides an explanation that the proliferation in the crypts was undisrupted by CT1113 treatment. Further, in the testis, CT1113 administration did not cause any aberrations in the histological organization nor in the proliferation of the spermatogonia (Supplementary Fig. 5c).

In summary, we identified a class of potent USP25/28 inhibitors that shows broad anti-tumor activity.

## Supplementary information

Supplementary_Materials -220921

## Data Availability

All data are available from the corresponding author on reasonable request.
