# Supplementary material for: Identification of a class of potent USP25/28 inhibitors with broad-spectrum anti-cancer activity
Source: Signal Transduct Target Ther. 2022 Dec 8;7:393. doi: 10.1038/s41392-022-01209-2 (PMC9731946; doi:10.1038/s41392-022-01209-2)
Supplement: Supplementary file 1 — Supplementary_Materials -220921 [file 41392_2022_1209_MOESM1_ESM.docx]

Supplementary Materials for

**Identification of A Class of Potent USP25/28 Inhibitors With**

**Broad-spectrum Anti-cancer Activity**

Jin Peng ^1, #^, Kun Jiang ^2, #^, Xiao Sun ^1^, Lingzhi Wu ^1^, Jiewei Wang ^2^, Xiaomei Xi ^3^, Xu Tan ^4^,
Tingbo Liang ^1, *^, Changheng Tan ^2, *^ and Pumin Zhang ^1, *^

^1^ Zhejiang Provincial Key Laboratory of Pancreatic Diseases, The First Affiliated Hospital of Zhejiang University, Hangzhou, Zhejiang 310003, China

^2^ Shanghai Institute of Materia Medica, Shanghai 200216, China

^3^ Chaser Therapeutics, Hangzhou, Zhejiang Province 310018, China

^4^ Beijing Advanced Innovation Center for Structural Biology, School of Pharmaceutical Sciences, Tsinghua-Peking Center for Life Sciences, Tsinghua University, Beijing, China

^#^ These authors contributed equally to this work.

* Corresponding authors

Correspondence to: Pumin Zhang, Ph.D. ([pzhangbcm@zju.edu.cn](mailto:pzhangbcm@zju.edu.cn))

**This PDF file includes:**

Materials and Methods

Figures. S1 to S5

Tables S1 to S4

**Materials and Methods**

*DUB activity assay*

Dilute DUBs to appropriate concentrations in the DUB assay buffer (50 mM Tris HCl pH 7.5/1 mM EDTA/100 mM NaCl/0.05% CHAPS/5 mM DTT), add ubiquitin-rhodamine 110 (or other substrates) to the final concentration of 125 nM, and immediately start serial reading on a plate reader. To measure the rates of inhibition of compounds, the compound was first incubated with the DUB for 30 min before the addition of the substrate. The inhibition rate was calculated according to the following formula:

Inhibition rate=RFU_compound_ (time of reading)-RFU_substrate only_ (average of multiple timepoints)/RFU_DMSO_ (time of reading)-RFU _substrate only_ (average of multiple timepoints)

*Compound library screen and compound optimization*

The 100,000 compounds library from ChemBridge (San Diego, CA) was screened manually using the DUB assay. The concentration of the compounds used for the screen was 10 μM. Any compounds with an inhibition rate above 50% were re-screened. Conventional organic synthesis was employed to synthesize derivatives of the compounds from the screen (CT1001-1004) to obtain compounds with better inhibitory activities.

*Binding kinetics*

We used a benchtop SPR (surface plasma resonance) instrument from Nicoya to measure binding kinetics according to the instructions by the manufacturer. USP25 and USP28 protein were immobilized on special glass slide and the small molecule inhibitors were flowed through the slide for interaction with the immobilized protein.

*Cell culture, viral transduction and cell treatment*

The cancer cell lines were cultured in DMEM, RPMI-1640 or F12 Medium with 10% FBS in a humidified incubator containing 5% CO_2_ at 37 ^0^C. The plasmids used in the work were generated through standard cloning methods. Lentiviruses-carrying overexpression or knockdown elements were produced in the lab and used to infect the cell lines with MOI (multiplicity of infection) ＞1. The infected cells were selected with puromycin treatment (4 μg/ml for 2 days). To prevent proteasomal degradation, MDAH2774 cells were incubated with 10 μM MG132 for 6 hours following pretreatment with 500 nM CT1073 or CT1113. For cycloheximide chase analysis, MDA-MB-231 or HCT116 cells were treated with 500 nM CT1073 and 100 μg/ml cycloheximide for the indicated times before sample collection. To express *USP25* or *USP28* exogenously, HCT116 cells were infected with lentiviruses expressing GFP, HA-USP28 or HA-USP25 (constructed in pHAGE) and selected for with puromycin. To express the two together, the cells were infected with HA-USP25 viruses first and after puromycin selection, re-infected with HA-USP28 viruses. The second infection was not selected for but the number of viruses was ensured with a control infection in fresh HCT116 cells.

*Assays for cell proliferation*

For MTS assay, the cells were trypsinized and re-seeded in 96-well plates at a density of 2×10^3^ cells/well and cultured for the indicated times. At the end of incubation, the cell viability was analyzed using a colorimetric assay (MTS, Promega, Madison, WI). Briefly, 20 μl MTS was added to 100 μl fresh complete culture medium in each well, and incubated for 2 hrs before the absorbance of the formazan product at 490 nm was measured.

*Fluorescence activated cell sorting*

The cells were trypsinized and washed once with cold PBS. For cell cycle analysis, the cells were fixed in 70% ice-cold EtOH, spun down, washed with cold PBS, and incubated in PBS containing propidium iodide (PI, 50 μg/ml) and RNase A (50 μg/ml) for 30 min at room temperature. The PI-stained single cell suspension was analyzed on a BD LSRFortessa SORP Flow Cytometer (BD Biosciences). ModFit LT software (Verity Software House, Topsham, ME, USA) was used to analyze the DNA patterns and cell cycle stages.

For apoptosis assay, the cells were stained with AnnexinV-FITC and propidium iodide for 15~30 mins at room temperature prior to flow cytometry

*Western blotting analysis*

The cells or tissues were lysed in RIPA buffer (Applygen Technologies Inc., Beijing, China) supplemented with a protease inhibitor cocktail (Roche Diagnostics, Mannheim, Germany), and the lysates were centrifuged at high speed to remove insoluble debris. The protein concentration of the resultant lysates was determined with a bicinchoninic acid (BCA) assay kit (Beyotime, Shanghai, China). Equal amounts of proteins were boiled for 5 min in 5x SDS loading buffer (Biosharp, Hefei, China), separated in a SDS-polyacrylamide gel, and transferred onto nitrocellulose membranes. The membranes were incubated for 1 hr in blocking buffer (5% non-fat dry milk in TBST) and then with primary antibodies at 4°C overnight. After 3 washes with TBST, the membrane was incubated for 1 hr at room temperature with horseradish peroxidase (HRP)-conjugated secondary antibodies. The membrane was then washed three times and visualized with SuperSignal™ West Pico Chemiluminescent Substrate (Thermo Fisher Scientific, San Jose, CA, USA). The expression of GAPDH, Actin or Tubulin was routinely used as a loading control.

*Ubiquitination assay*

For c-MYC ubiquitination assay, MDA-MB-231 cells were treated with 500 nM CT1073 or CT1113 and 20 μM MG132 for 2 hrs. The cells were harvested and lysed in NETN buffer (pH8.0 tris-HCl, 100mM NaCl, 1 mM EDTA, 0.5% Nonidetp-40) containing 1% SDS and 1% sodium deoxycholate, vortexed vigorously for 15~30 minutes, boiled for 10 minutes, and then 5-9 times of the volume of NETN buffer were added. The cell lysates were then incubated with anti-c-MYC antibodies and proteinA/G agarose beads overnight at 4^o^C followed by washing and western blotting analysis of the immunoprecipitates.

*RNA-seq analysis*

Total RNA was isolated using TRIzol^®^ reagent from MDA-MB-231 cells treated with control (DMSO) or 500 nM CT1073 for 48 hrs and submitted to Lianchuan Biological Information Technology (Hangzhou, China) for library preparation, sequencing, and initial analysis. Gene set enrichment analysis was performed with an open software (https://www.gsea-msigdb.org/gsea/index.jsp).

*Tumor xenografts and CT1113 treatment*

For CDX (cell-derive xenograft), the tumor cells in logarithmic growth phase were collected by trypsin digestion, washed twice with PBS, resuspended in PBS, mixed with matrix gel in a ratio of 1:3 (matrix gel : cells), and placed on ice. The tumor cells (SW1990, 5 X 10^6^ and HCT116, 5 X 10^6^) were inoculated subcutaneously into nude mice within an hour of preparation. CT1113 treatment (20 mg/kg body weight, twice a day through an oral gavage) started once the tumor mass became palpable. At the end of treatment, the tumor-bearing animals were sacrificed, and the tumor mass dissected out for analysis.

To observe the effect of CT1113 on tissue renewal, male C57BL/6 mice were given the compound at 20 mg/kg body weight twice a day for 21 consecutive days followed by 21 days of recovery. During the course of the experiment, body weight was measured every other day. At end of 21-day treatment, 3 vehicle control and 3 CT1113 treated animals were sacrificed, and major organs dissected out for histological examination. 2 hours before the sacrifice, the animals were intraperitoneally injected with bromodeoxy uridine (BrdU, dissolved in PBS at 1 mg/ml) at a dose of 25 μg/g body weight.

All animal experiments were performed according to the guidelines approved by the Animal Care and Use Committee of the First Affiliated Hospital of Zhejiang University.

*Histology and Immunohistochemical staining*

Tissues were fixed in 10% formaldehyde at 4^o^C overnight. Tissue processing, sectioning, and histological and immunohistochemical stainings were performed by Houke Biotech (Hangzhou, China) and Xiangyou Tech (Shanghai, China).

*Statistical analysis*

Statistics analyses were performed with GraphPad Prism9.0 and ImageJ. Unpaired two-tailed Student’s *t* test was used to analyze the significance between two groups. P<0.05 was considered as statistical significance (*, p<0.05; **, p<0.01; ***, p<0.001, ****, p<0.0001).


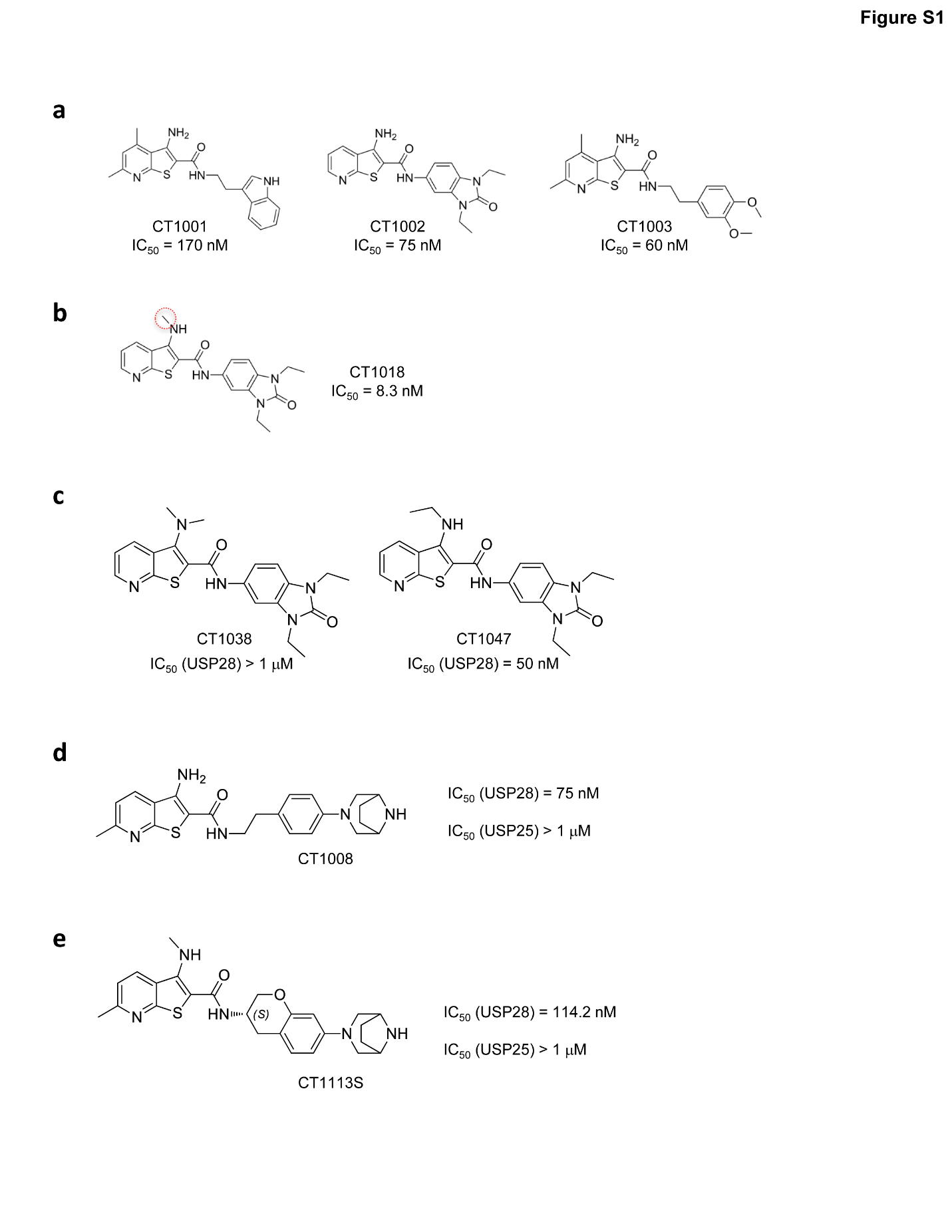


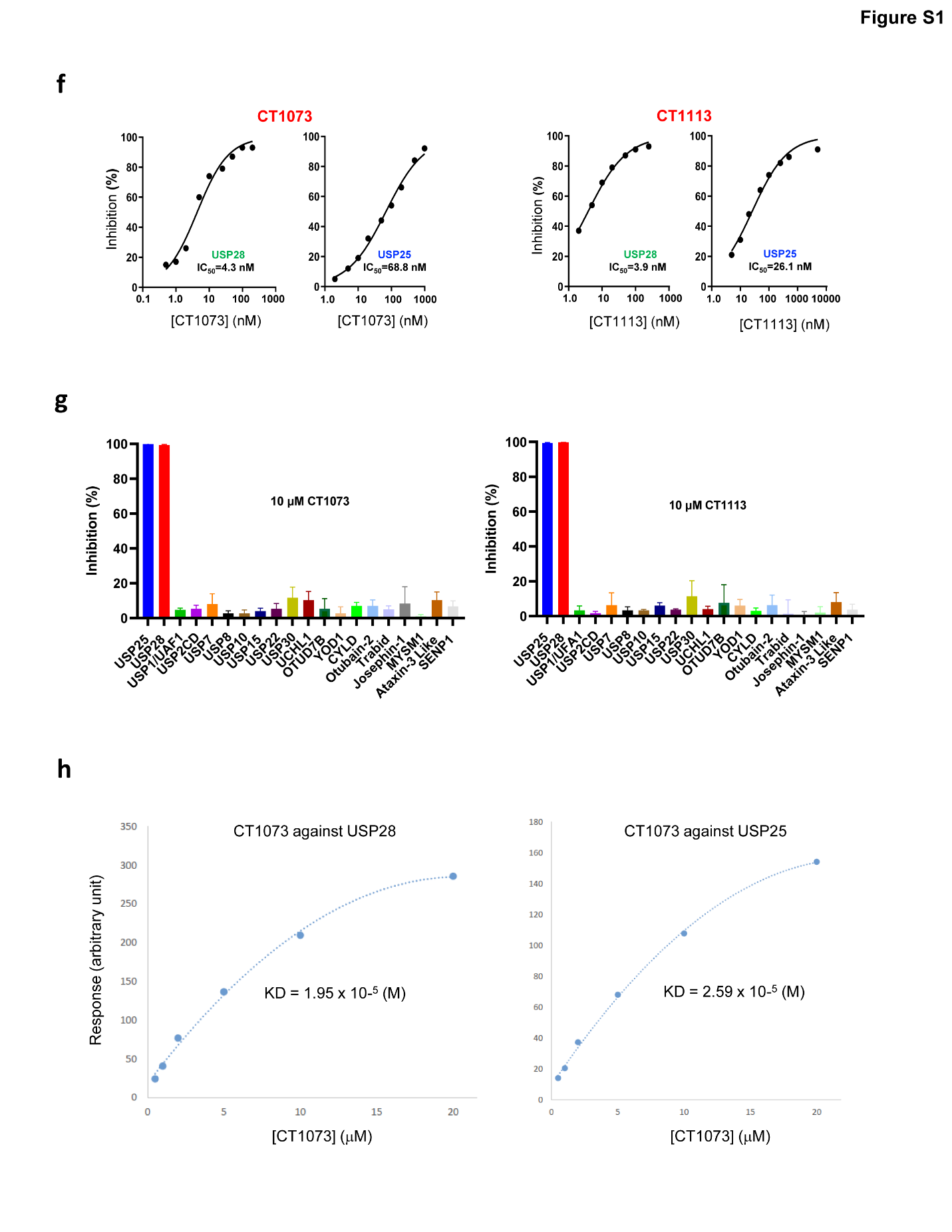


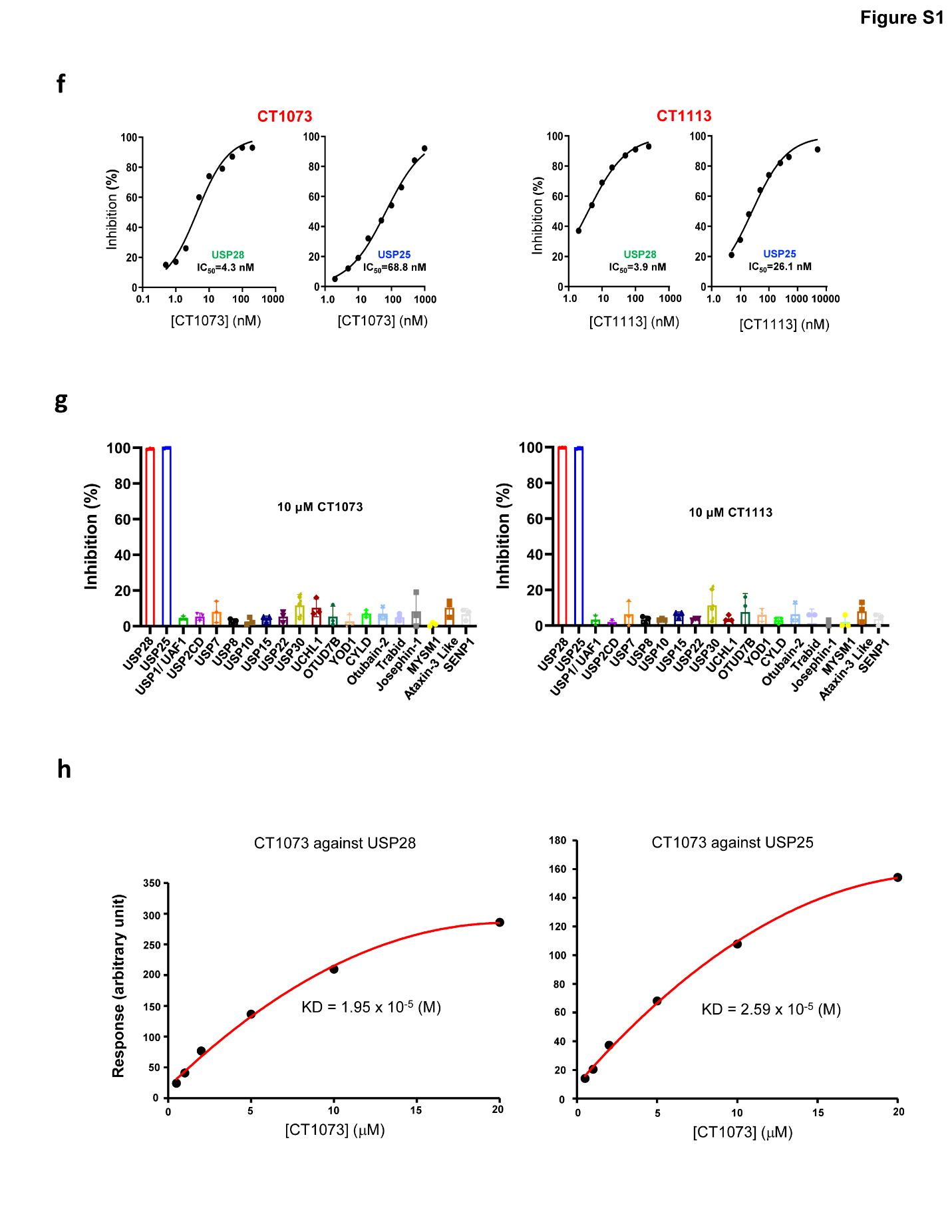


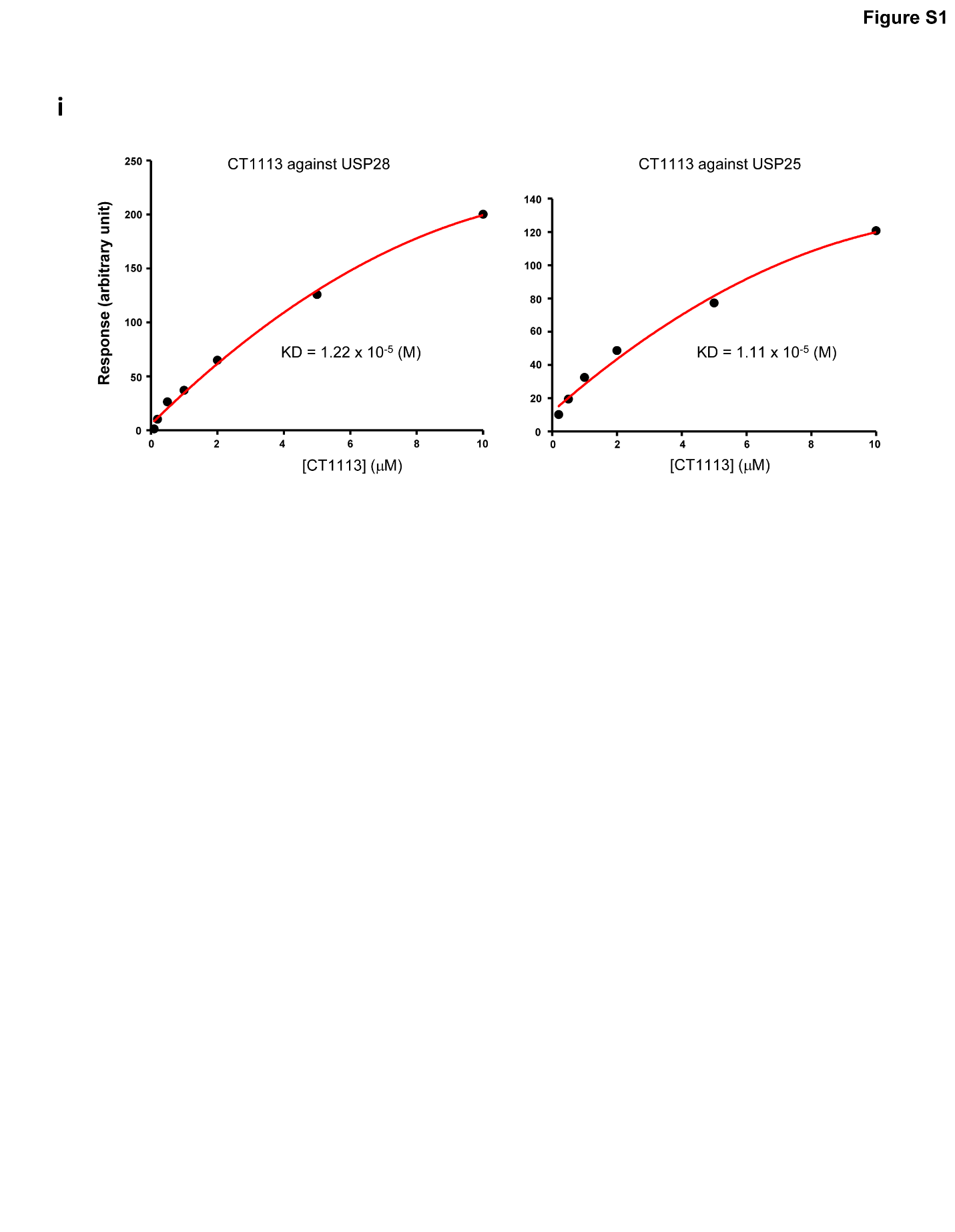


**Supplementary Fig. 1. Chemical Structures of additional USP28 inhibitors**

**a.** The chemical structures and IC_50_s of CT1001-1003 identified from a library of 100,000 synthetic compounds. The library was screened manually in a 384-well format. Each well contains a reaction mix of USP28 (final concentration 4 nM) and Rhodamine-ubiquitin (final concentration 125 nM) in a buffer.

**b.** The chemical structure and IC_50_ of CT1018. The red dashed circle highlights the semi-methylation not present in CT1002.

**c.** The chemical structures and IC_50_s of CT1038 and CT1047.

**d.** The chemical structure and IC_50_s of CT1008.

**e.** The chemical structure and IC_50_s of CT1113S.

**f.** IC_50_s determination of CT1073 and CT1113.

**g.** The inhibitory activities of 10 μM CT1073 and CT1113 against other deubiquitinases and SENP1. Each measurement was performed 3 or more times and the data are mean ± SD.

**h, i.** The interaction kinetics between USP25/28 and CT1073 (**h**) or CT1113 (**i**) were measured with an SPR instrument. KD values are shown.


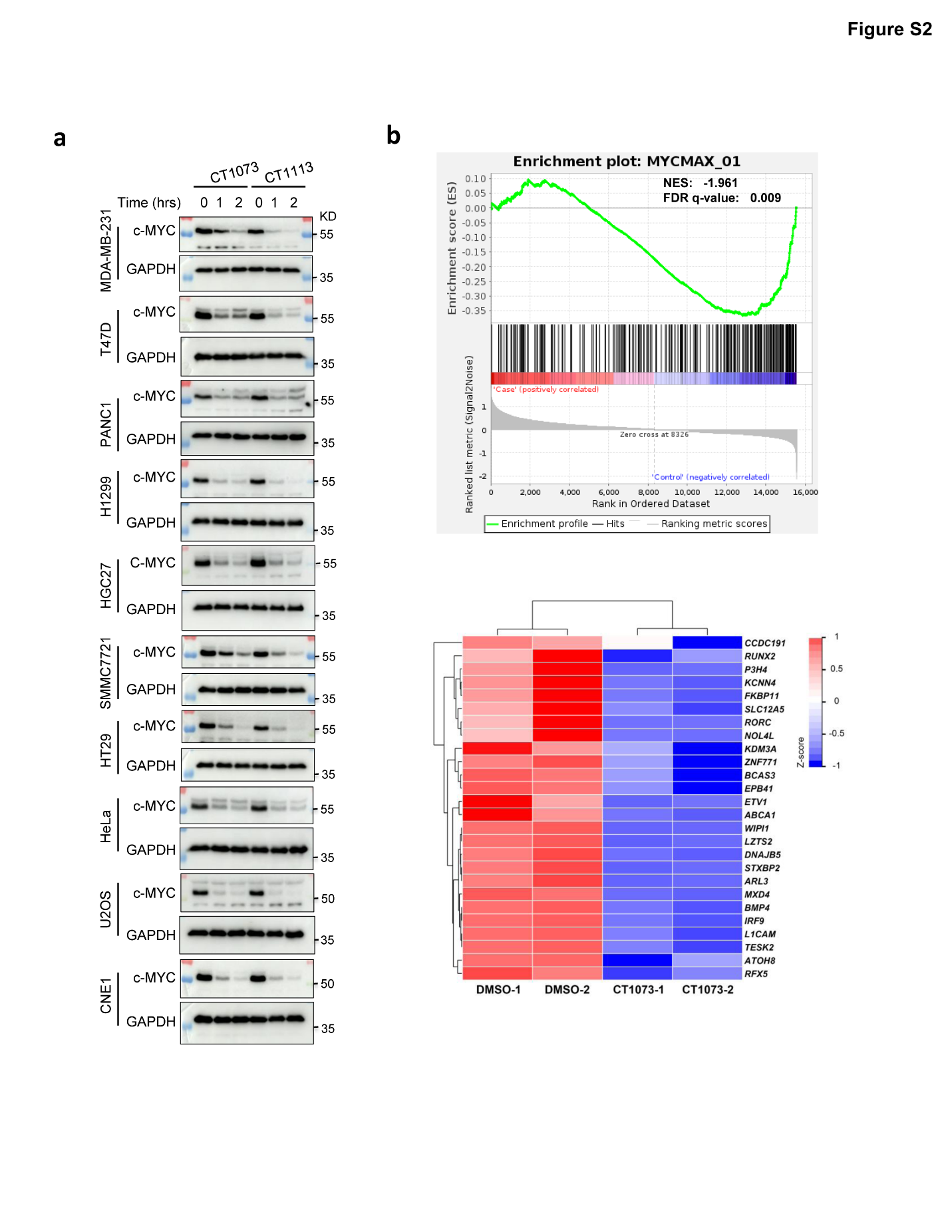


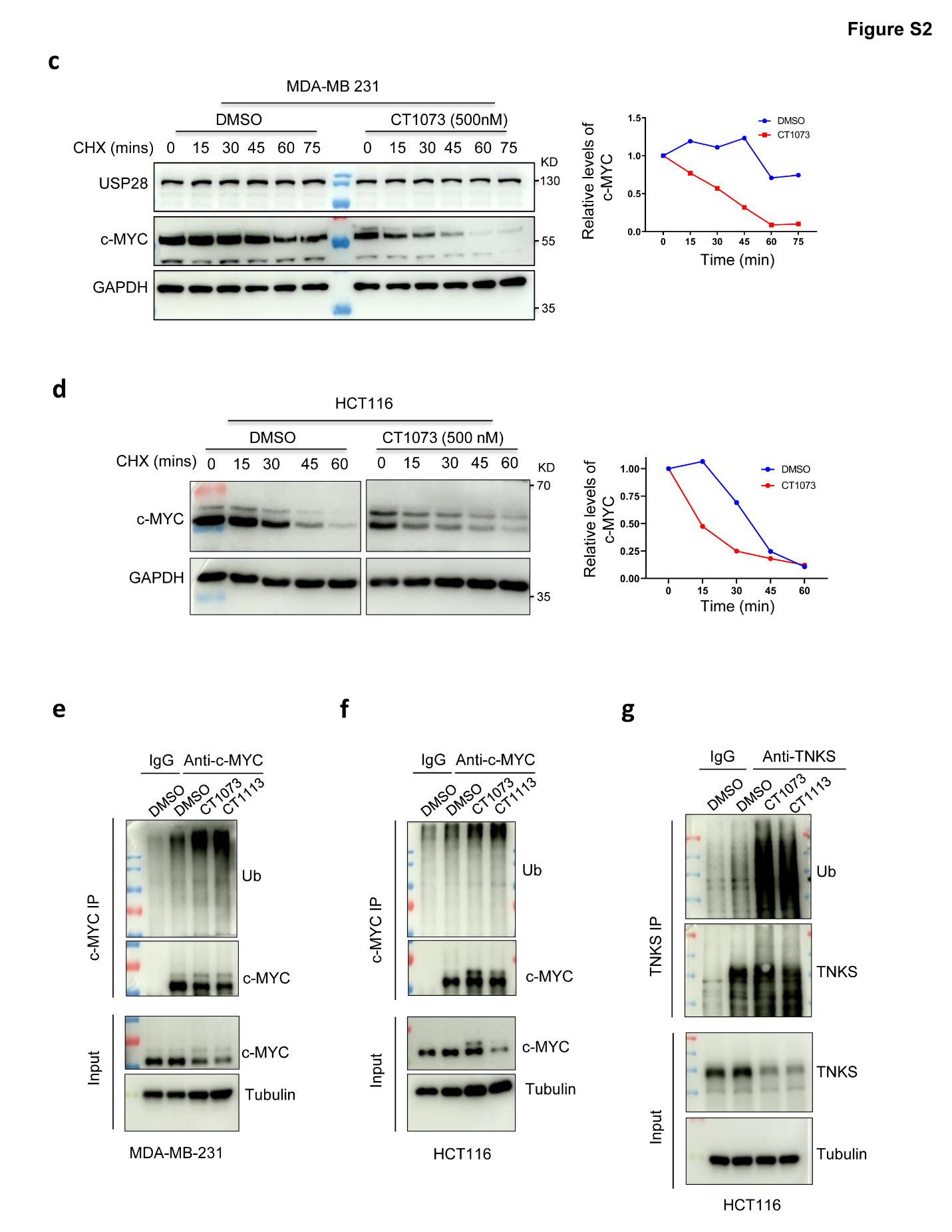


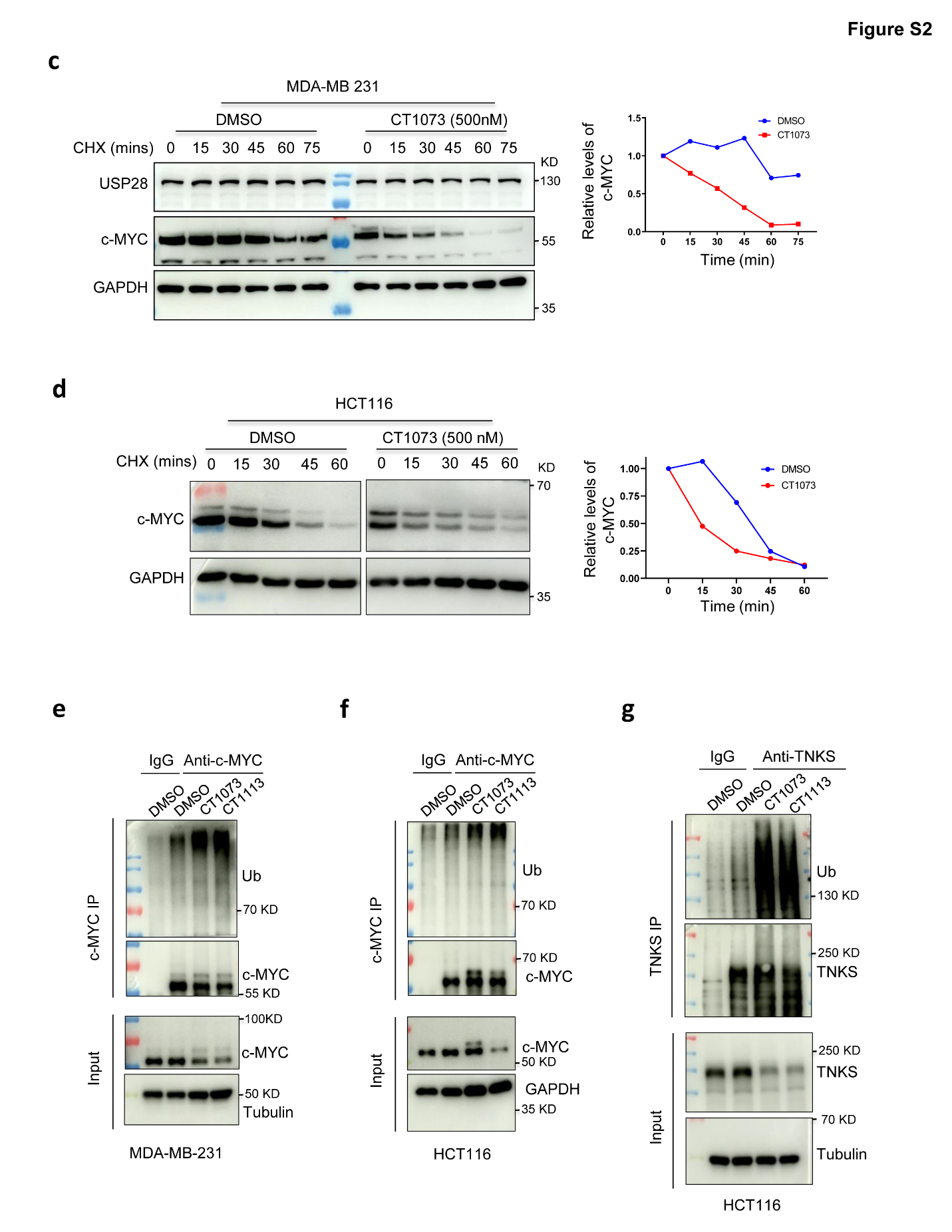


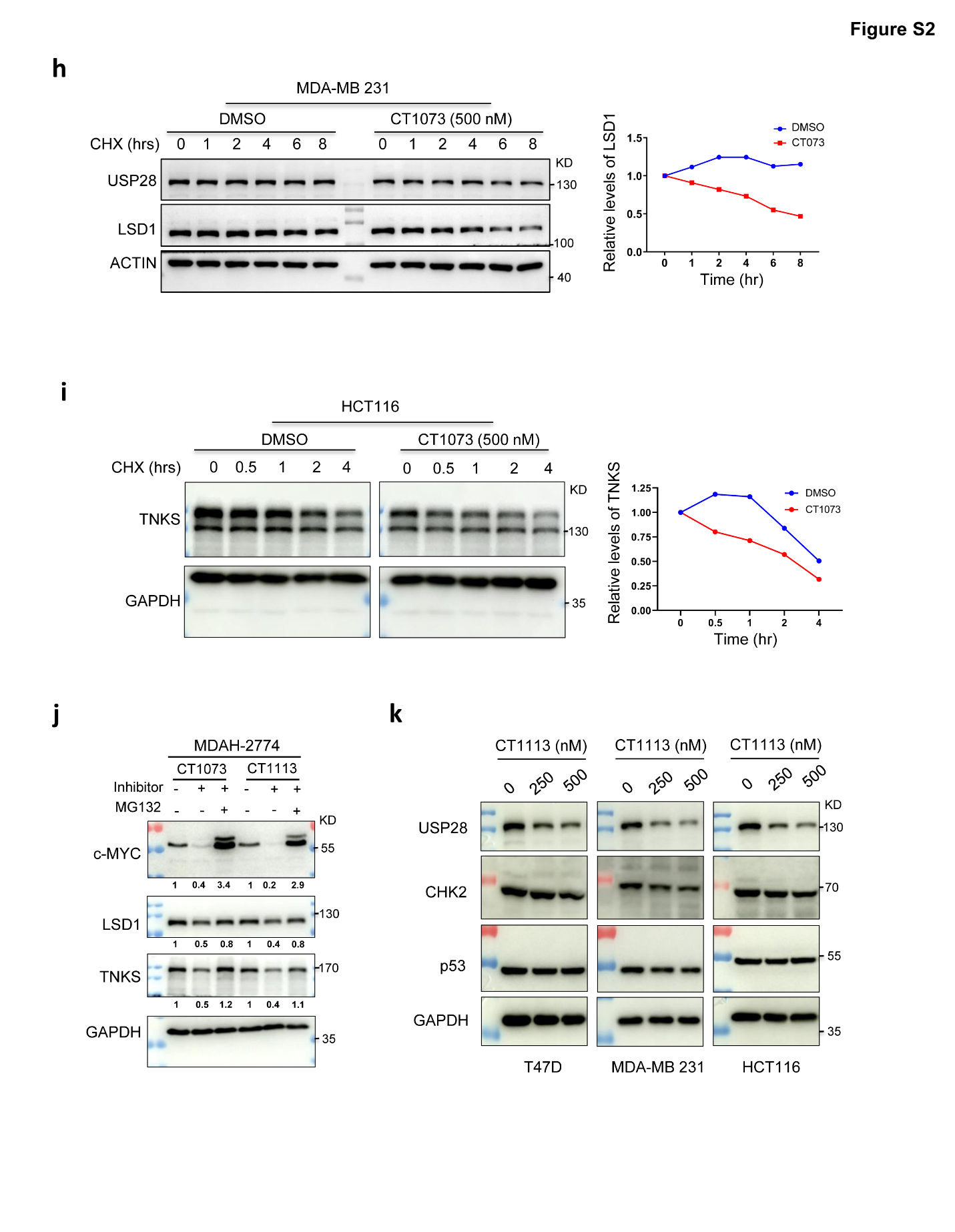


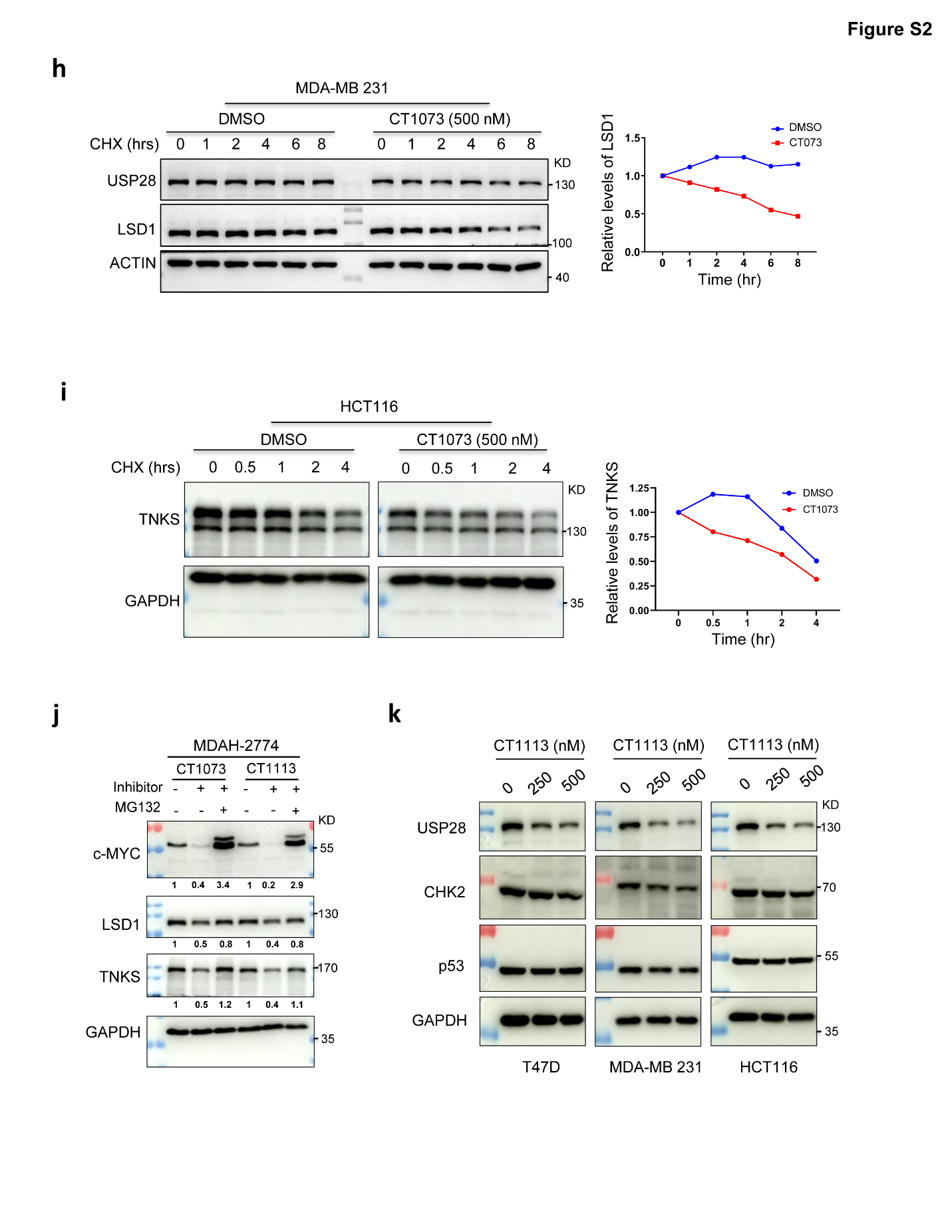


**Supplementary Fig. 2.**  **Inhibiting USP28 and USP25 in cells**

**a.** Western blotting analysis of c-MYC in the cells treated with 500 nM CT1073 or CT1113.

**b.** RNA-seq analysis of MDA-MB-231 cells treated with 500 nM CT1073 for 2 days. MYC-MAX target genes were subjected to GESA analysis and the top 26 affected genes were presented in a heatmap. FDR, false discovery rate; NES, normalized enrichment score.

**c, d.** Cycloheximide-chasing experiment to determine the half-life of c-MYC in control and CT1073-treated MDA-MB-231 (**c**) and HCT116 (**d**) cells. The cells were pre-treated with CT1073 for 2 hrs, then treated with 100μg/ml cycloheximide (CHX), and harvested at different time points for analysis.

**e, f.** The ubiquitination assay of c-MYC in MDA-MB-231 (**e**) and HCT116 (**f**) cells treated with 500 nM CT1073 or CT1113 and MG132 (20 μM) for 2 hrs.

**g.** The ubiquitination assay of Tankyrase in HCT116 cells treated with 500 nM CT1073 or CT1113 and MG132 (10 μM) for 8 hrs.

**h, i.** Cycloheximide-chasing experiment to determine the half-life of LSD1 (**h**) and Tankyrase (**i**) in control and CT1073-treated cells.

**j.** Western blotting analysis of the indicated proteins in MDAH-2774 cells treated with CT1073 or CT1113 for 7 hrs then plus or minus MG132 (10 μM) for 6 hrs.

**k.** Western blotting analysis of p53 and CHK2 in the cells treated with CT1113 for 48 hrs.


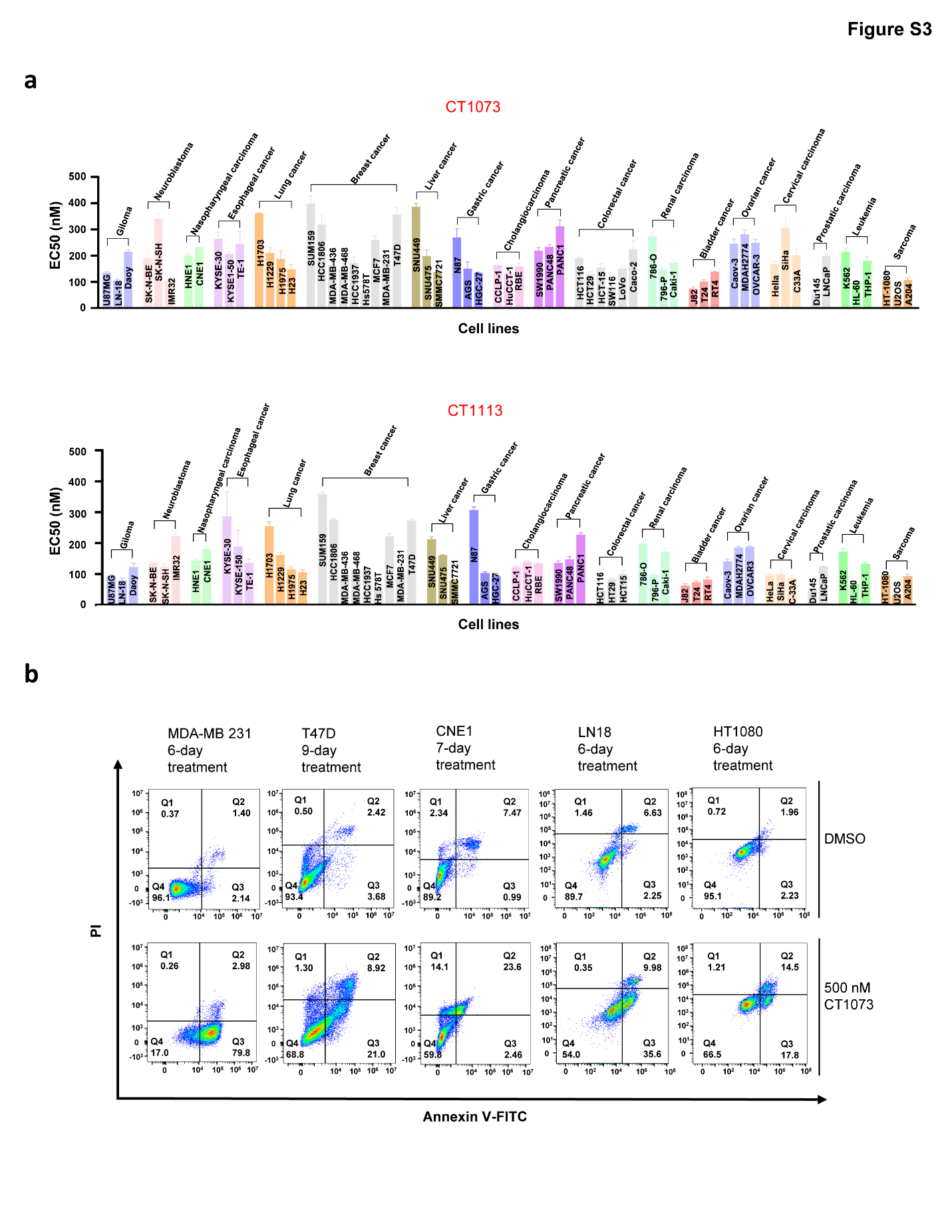


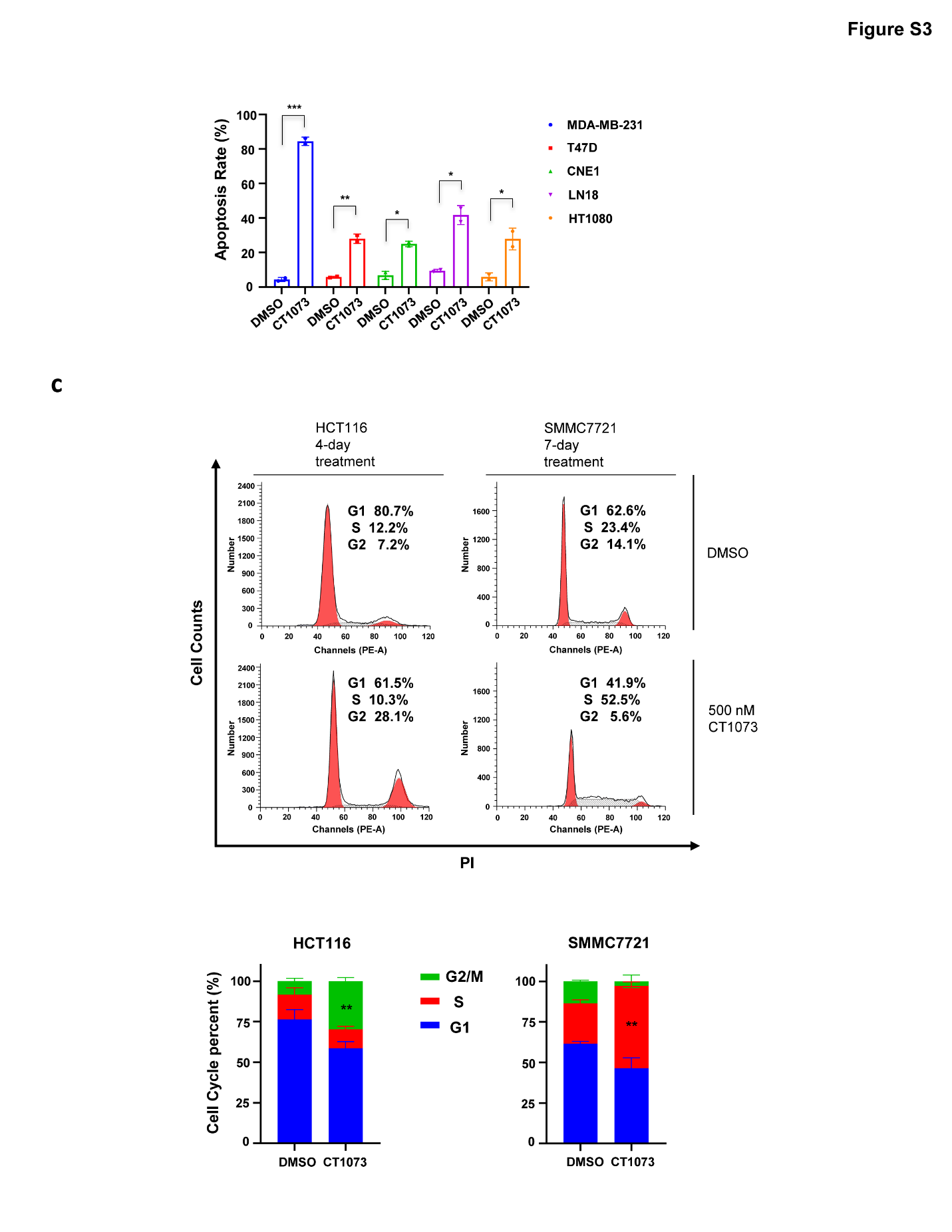


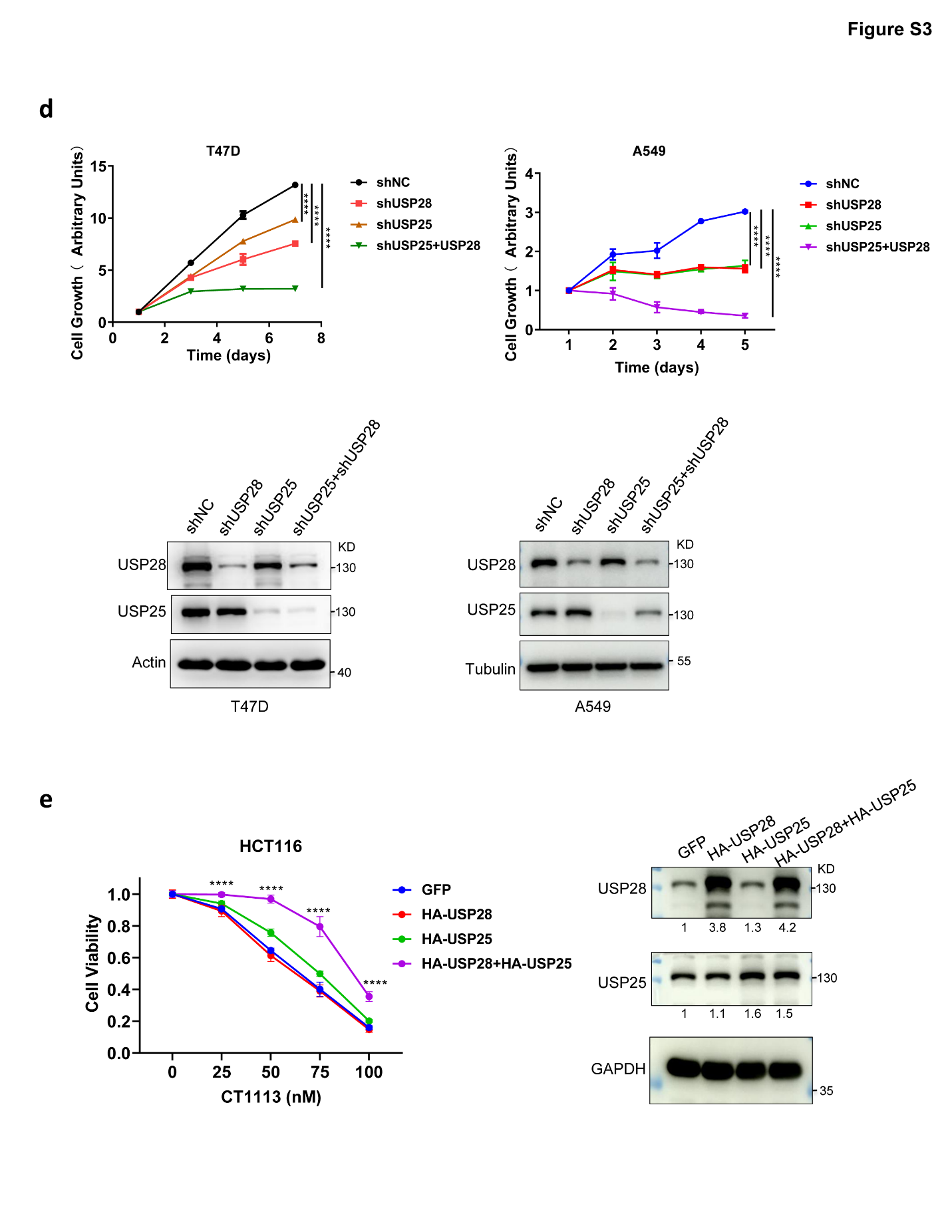


**Supplementary Fig. 3.** **The effects of inhibiting USP28 and USP25 in cells**

**a.** EC_50_s of CT1073 and CT1113 against the cancer cell lines derived from major tumor types. The cells were treated with the compound of different concentrations for 72 hrs and the viability of the cells were determined with the MTS assay. Each EC50 determination was performed 3 or more times and the data are mean ± SD.

**b.** FACS analysis of apoptosis in the cells treated with CT1073. The data were analyzed by the FlowJo software, and the apoptosis rate was calculated. Student’s *t* test: *, p<0.05; **, p<0.01; ***, p<0.001.

**c.** Cell cycle distribution in HCT116 and SMMC7721 cells treated with CT1073. The percentage of the cells in each cell cycle phase were calculated with ModFit LT software. Phase percentages for G1, S, and G2/M are displayed by bar graph. Student’s *t* test: **, p<0.01.

**d.** Cell growth analyses. T47D and A549 cells were depleted of *USP28, USP25* or both through shRNAs and the viability of the cells were determined with the MTS assay. The experiment was performed 3 times and the data are mean ± SD. Student’s *t* test: ****, p<0.0001.

**e.** Cell growth analysis of the HCT116 cells expressing exogenous *USP25* and *USP28.* Data from 3 days of culturing were plotted. The experiment was performed 3 times and the data are mean ± SD. Student’s *t* test: ****, p<0.0001. Western blotting was used to assess the levels of expression. *USP28* could be expressed to a much higher level than *USP25*.


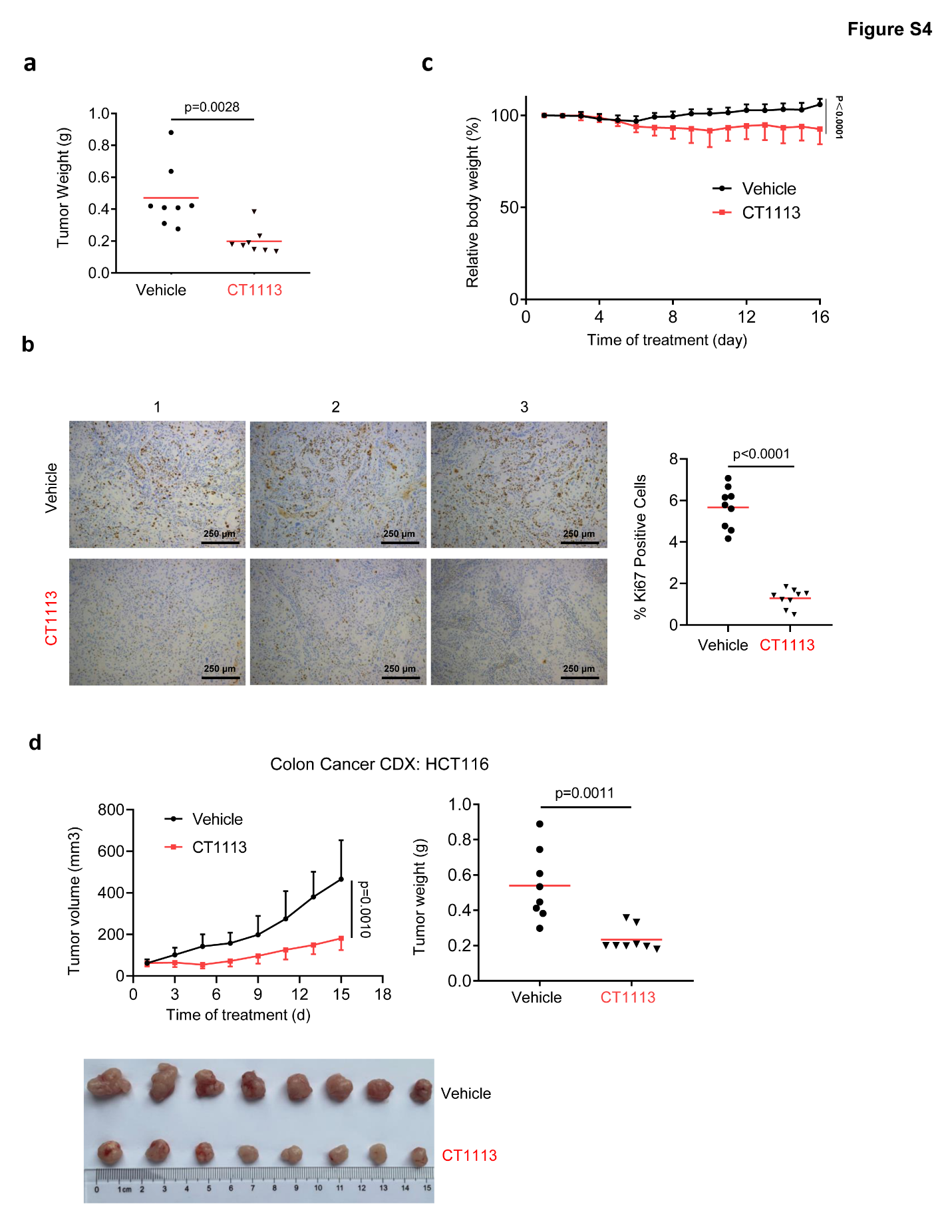


**Supplementary Fig. 4.** **CT1113 suppresses tumor growth in mice**

**a.** The weight of the tumors in **Fig. 1c** are presented (n = 8 mice per group).

**b.** Immunohistochemical staining of Ki67 in the tumor samples from **Fig. 1c**. 3 samples were selected from control and the treatment group, and three random microscopic fields were imaged from each sample. The staining intensity was determined with Image J software and plotted.

**c.** The body weight of the tumor (SW1990)-bearing mice. Two-tailed Student’s tests were performed to examine the statistical significance.

**d.** The effect of CT1113 in a colon cancer CDX model generated by inoculating HCT116 cells into Balb/c nude mice (n = 8 mice per group). The growth curve, the weight and the photographs of the tumors are presented. Two-tailed Student’s tests were performed to examine the statistical significance.


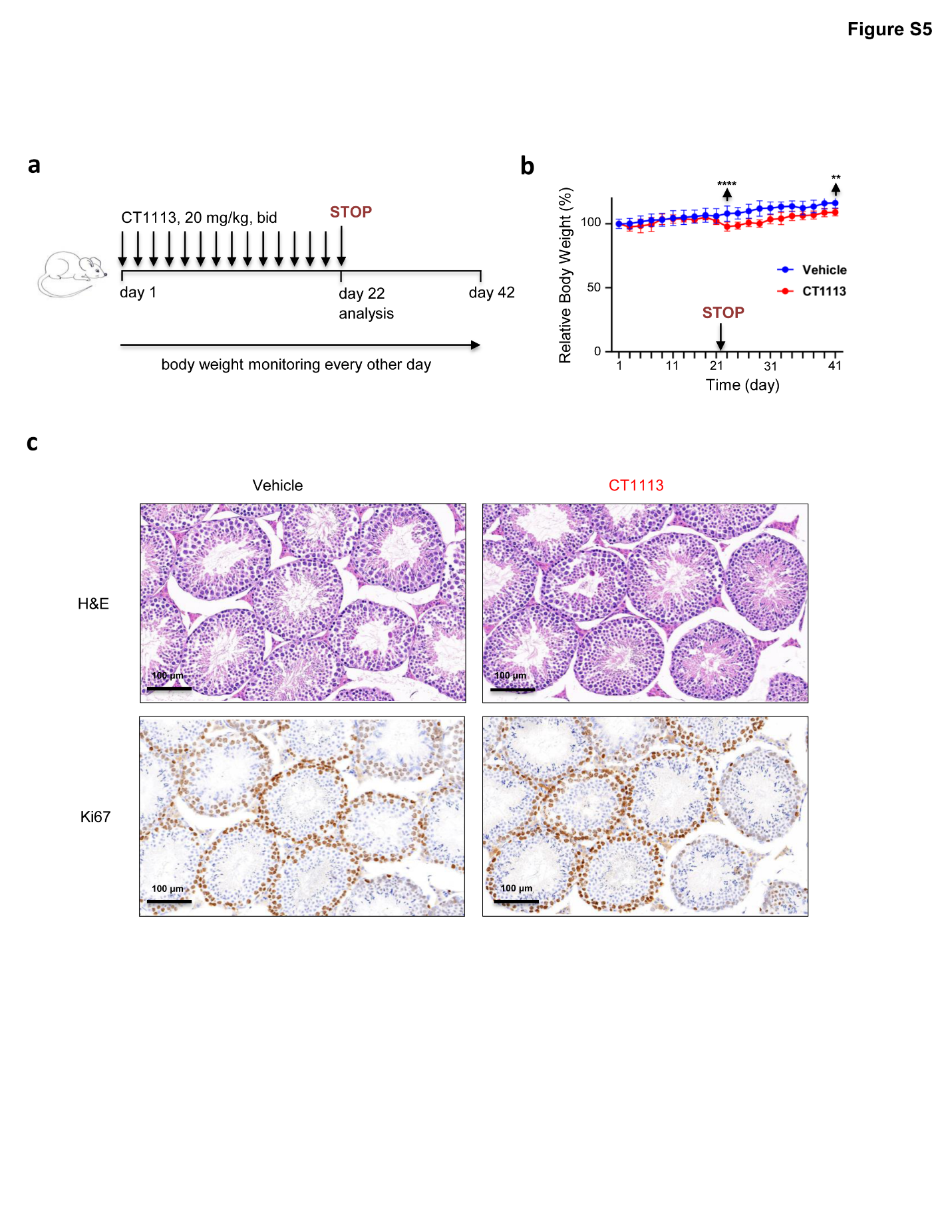


**Supplementary Fig. 5.** **The effect of inhibiting USP28 and USP25 on regenerating tissues in mice**

**a.** The timeline of CT1113 treatment of male C57BL/6 mice (n = 3 mice per group).

**b.** The body weight of the mice over the course of the treatment and the subsequent recovery period. The data are mean ± SD. Student’s *t* test: **, p<0.01; ****, p<0.0001.

**c**. Hematoxylin and eosin (H&E) staining and Ki67 immunohistochemical staining of the testis sections from control and CT1113-treated (3 weeks) mice.

**Table S1. Cell lines**

| **Cell lines** | **SOURCE** |
| --- | --- |
| 786-O | ATCC |
| 796-P | ATCC |
| A204 | COBIOER |
| A549 | kindly provided by Cell Bank/Stem Cell Bank, Chinese Academy of Sciences |
| AGS | Procell |
| C-33A | kindly provided by Cell Bank/Stem Cell Bank, Chinese Academy of Sciences |
| Caco2 | ATCC |
| CaKi-1 | ATCC |
| Caov-3 | kindly provided by Cell Bank/Stem Cell Bank, Chinese Academy of Sciences |
| CCLP1 | kindly provided by Zhejiang Provincial Key Laboratory of Pancreatic Disease |
| CNE1 | kindly provided by Cancer Center, Shanghai General Hospital, Shanghai Jiao Tong University School of Medicine |
| Daoy | kindly provided by Cell Bank/Stem Cell Bank, Chinese Academy of Sciences |
| DU145 | ATCC |
| H1229 | ATCC |
| H1703 | ATCC |
| H1975 | ATCC |
| H23 | kindly provided by Cell Bank/Stem Cell Bank, Chinese Academy of Sciences |
| HCC1806 | ATCC |
| HCC1937 | ATCC |
| HCT116 | kindly provided by Cell Bank/Stem Cell Bank, Chinese Academy of Sciences |
| HCT15 | ATCC |
| HeLa | ATCC |
| HGC-27 | COBIOER |
| HL-60 | kindly provided by Department of Hemotology,The First Affiliated Hospital, Zhejiang University School of Medicine |
| HNE-1 | kindly provided by Cancer Center, Shanghai General Hospital, Shanghai Jiao Tong University School of Medicine School of Medicine |
| Hs578T | ATCC |
| HT-1080 | COBIOER |
| HT29 | ATCC |
| HuCCT1 | kindly provided by Zhejiang Provincial Key Laboratory of Pancreatic Disease |
| IMR32 | kindly provided by Cell Bank/Stem Cell Bank, Chinese Academy of Sciences |
| J82 | COBIOER |
| K562 | kindly provided by Department of Hemotology, The First Affiliated Hospital, Zhejiang University School of Medicine |
| KYSE-150 | Procell |
| KYSE-30 | Procell |
| LN-18 | COBIOER |
| LNCaP | COBIOER |
| LoVo | ATCC |
| MCF7 | ATCC |
| MDA-MB-436 | ATCC |
| MDA-MB-231 | ATCC |
| MDA-MB-468 | ATCC |
| MDAH2774 | ATCC |
| NCI-N87 | kindly provided by Cell Bank/Stem Cell Bank, Chinese Academy of Sciences |
| NIH: OVCAR-3 | kindly provided by Cell Bank/Stem Cell Bank, Chinese Academy of Sciences |
| PANC1 | ATCC |
| PANC48 | kindly provided by Zhejiang Provincial Key Laboratory of Pancreatic Disease |
| RBE | kindly provided by Cell Bank/Stem Cell Bank, Chinese Academy of Sciences |
| RT4 | COBIOER |
| SiHa | ATCC |
| SK-N-BE | kindly provided by Cell Bank/Stem Cell Bank, Chinese Academy of Sciences |
| SK-N-SH | kindly provided by Cell Bank/Stem Cell Bank, Chinese Academy of Sciences |
| SMMC7721 | Thermo Fisher Scientific |
| SNU449 | ATCC |
| SNU475 | ATCC |
| SUM159 | COBIOER |
| SW116 | kindly provided by Zhejiang Provincial Key Laboratory of Pancreatic Disease |
| SW1990 | ATCC |
| T24 | COBIOER |
| T47D | ATCC |
| TE-1 | kindly provided by Cell Bank/Stem Cell Bank, Chinese Academy of Sciences |
| THP-1 | kindly provided by Department of Gastroenterology, The First Affiliated Hospital of Zhejiang University School of Medicine |
| U-87MG | COBIOER |
| U2OS | ATCC |

**Table S2. Reagents**

| **Reagents** | **SOURCE** | **IDENTIFIER** |
| --- | --- | --- |
| Recombinant Human His6-USP25 Protein | Boston Biochem | E546 |
| Recombinant Human His6-USP28 Protein | Boston Biochem | E570 |
| Recombinant Human Ubiquitin Rhodamine 110 Protein | Boston Biochem | U555 |
| Recombinant Human His6-USP1/His6-UAF1 Complex Protein | Boston Biochem | E568 |
| Recombinant Human USP2 Catalytic Domain Protein | Boston Biochem | E504 |
| Recombinant Human His6-USP7 Protein | Boston Biochem | E519 |
| Recombinant Human His6-USP8 Protein | Boston Biochem | E520 |
| Recombinant Human His6- USP10 Protein | Boston Biochem | E592 |
| Recombinant Human His6-USP22 Protein | Boston Biochem | E608 |
| Recombinant Human His6-USP30 Protein | Boston Biochem | E582 |
| Recombinant Mouse His6-UCH-L1 Protein | Boston Biochem | E335 |
| Recombinant Human His6-USP15 Protein | Boston Biochem | E594 |
| Recombinant Human His6- OTUD7B Protein | Boston Biochem | E562 |
| Recombinant Human His6- YOD1 Protein | Boston Biochem | E572 |
| Recombinant Human His6- CYLD Isoform 1 Protein | Boston Biochem | E556 |
| Recombinant Human His6-Otubain-2 Isoform 1 Protein | Boston Biochem | E554 |
| Recombinant Human His6-ZRANB1/Trabid Protein | Boston Biochem | E560 |
| Recombinant Human His6-Josephin-1 Protein | Boston Biochem | E618 |
| Recombinant Human MYSM1 Protein | Boston Biochem | E598 |
| Recombinant Human Ataxin-3 Like Protein | Boston Biochem | E343 |
| Recombinant Human Tetra Ub (k63 linked) Rhodamine 111 Protein | Boston Biochem | UC355 |
| Recombinant Human SUMO1 AMC Protein | Boston Biochem | UL551 |
| CellTiter 96® AQueous One Solution Cell Proliferation Assay | Promega | G3581 |

**Table S3. Antibodies**

| **Antibodies** | **SOURCE** | **IDENTIFIER** |
| --- | --- | --- |
| Anti-ACTIN | Proteintech | 66009-1-1g |
| Anti-BLM | Bethyl | A300-110A |
| Anti-BrdU | Proteintech | 66241-1-Ig |
| Anti-c-MYC | Genetex | GTX103436 |
| Anti-c-MYC | Proteintech | 10828-1-AP |
| Anti-c-MYC | Santa Cruz | SC-40 |
| Anti-GAPDH | Proteintech | 60004-1-1g |
| Anti- Tubulin | Proteintech | 66240-1-Ig |
| Anti-Ki67 | Abcam | ab16667 |
| Anti-LSD1 | Cell Signaling Technology | 2139S |
| Anti-MEK1/2 | Cell Signaling Technology | 4694S |
| Anti-RECQL1 | Bethyl | A300-450A |
| Anti-RECQL4 | Proteintech | 17008-1-AP |
| Anti-RECQL5 | Bethyl | A302-520A |
| Anti-TNKS | Proteintech | 18030-1-AP |
| Anti-Ub | Santa Cruz | SC-8017 |
| Anti-USP25 | Abclonal | A7975 |
| Anti-USP28 | Proteintech | 17707-1-AP |
| Anti-WRN | Cell Signaling Technology | 4666S |
| Anti-CHEK2 | Proteintech | 13954-1-AP |
| Anti-p53 | Santa Cruz | SC-126 |

**Table S4. shRNA sequences**

| **shRNA** | **sequence** |
| --- | --- |
| shNC | 5’-TTCTCCGAACGTGTCACGT-3’ |
| shUSP28-1 | 5’-GCACAGAAGTTCGTTGTCATA-3’ |
| shUSP28-2 | 5’-GACTGAAGATCATCCATTAAT-3’ |
| shUSP28-3 | 5’-AAGTGGCATGAAGATTATAGT-3’ |
| shUSP25-1 | 5’-GCGTGAGCTGAGGTATCTATT-3’ |
| shUSP25-2 | 5’- GCACTTCTCCTGTTGACGATA -3’ |
